# Interactive Effects of UV-B Light with Abiotic Factors on Plant Growth and Chemistry, and Their Consequences for Defense against Arthropod Herbivores

**DOI:** 10.3389/fpls.2017.00278

**Published:** 2017-03-02

**Authors:** Rocio Escobar-Bravo, Peter G. L. Klinkhamer, Kirsten A. Leiss

**Affiliations:** Plant Sciences and Natural Products, Institute of Biology of Leiden, Leiden UniversityLeiden, Netherlands

**Keywords:** blue light, drought, far-red light, herbivores, photosynthetically active radiation, plant defenses, temperature, ultraviolet-B light

## Abstract

Ultraviolet-B (UV-B) light plays a crucial role in plant–herbivorous arthropods interactions by inducing changes in constitutive and inducible plant defenses. In particular, constitutive defenses can be modulated by UV-B-induced photomorphogenic responses and changes in the plant metabolome. In accordance, the prospective use of UV-B light as a tool to increase plant protection in agricultural practice has gained increasing interest. Changes in the environmental conditions might, however, modulate the UV-B -induced plant responses. While in some cases plant responses to UV-B can increase adaptation to changes in certain abiotic factors, UV-B-induced responses might be also antagonized by the changing environment. The outcome of these interactions might have a great influence on how plants interact with their enemies, e.g., herbivorous arthropods. Here, we provide a review on the interactive effects of UV-B and light quantity and quality, increased temperature and drought stress on plant biochemistry, and we discuss the implications of the outcome of these interactions for plant resistance to arthropod pests.

## Introduction

As sessile organisms, plants can respond to simultaneous or sequential changes in abiotic conditions by modulating their physiology and, consequently, chemistry. Plant adaptive responses to external variations in growing conditions can have a profound effect on their responses to biotic stresses (Gouinguené and Turlings, 2002; Goel et al., 2008; Gutbrodt et al., 2011; Nguyen et al., 2016). In particular, light exerts a great impact on how plants are protected against herbivores or pathogens (reviewed by Ballaré, 2014). Light can be used as a powerful tool to increase plant resistance against herbivorous arthropods and, eventually, plant yield. Accordingly, for many crop species, manipulation of light conditions in greenhouses have become a common technique used by growers to increase plant performance, or to control photomorphogenic processes such as flowering (see for a review, Vänninen et al., 2010). In this regard, the prospective use of the ultraviolet-B (UV-B) light component of the solar radiation to enhance crop protection against pests and pathogens, as well as crop production, has gained increasing interest (Wargent and Jordan, 2013).

Ultraviolet-B (UV-B) light (280–315 nm) constitutes only a small fraction of solar radiation reaching the Earth’s surface. It represents, however, a crucial light signal to which plants can respond and develop specific photomorphogenic responses (Jenkins, 2009; Robson et al., 2015). Among these responses, changes in the morphology, physiology, and production of secondary metabolites are commonly described. The UV-B specific photoreceptor UV RESISTANT LOCUS (UVR8) regulates these photomorphogenic responses by controlling the expression of genes involved in the inhibition of hypocotyl elongation, DNA repair, antioxidative defense, and production of phenolic compounds that can act as UV-screening molecules (Rizzini et al., 2011). In order to reduce the oxidative damage and the penetration of UV light to photosynthetic cell layers, plants can accumulate flavonoids and phenylpropanoids in the leaf epidermis, and in both the palisade and spongy mesophyll tissues (Mazza et al., 2000; Agati et al., 2013).

**FIGURE 1:**
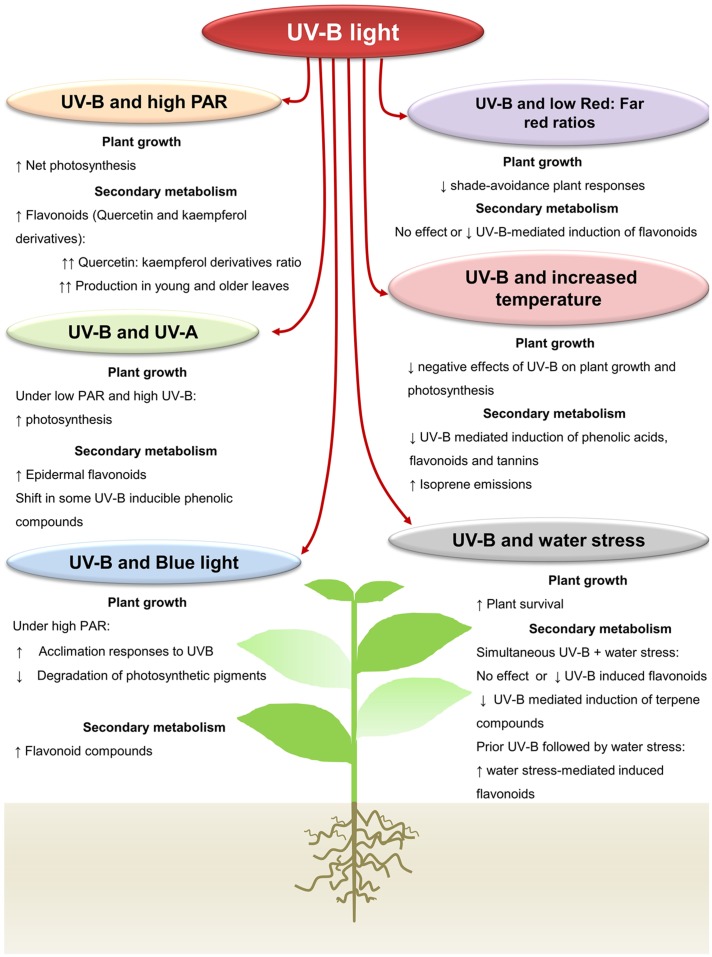
**Interactive effects of UV-B light with other abiotic factors on plant growth and production of plant secondary metabolites.** Under high Photosynthetic active radiation, UV-B light increases the net plant photosynthesis in several plant species. Higher production of flavonoids can be induced under both UV-B and high PAR in young and old plant leaves. UV-A radiation has a positive effect on the photosynthesis when plants are exposed to UV-B. Higher epidermal flavonoids are detected in plants under both UV-A and B radiations in some plant species. Exposition of plants to blue light prior or subsequent to UV-B also increases the acclimation responses to UV-B by reducing the degradation of photosynthetic pigments. Antagonistic responses between UV-B radiation and low-Red:far-red ratios have been reported. UV-B can inhibit the shade avoidance associated responses under low-Red:far-red ratios. Likewise, a low-Red:far red ratio can reduce the UV-B-mediated induction of plant flavonoids. Increased temperature increases acclimation of plants to UV-B, though it can reduce the UV-B-mediated induction of plant phenolics. Under combined UV-B radiation and increased temperature, however, higher emission of the plant volatile isoprene can be detected in some plant species. Similarly, under UV-B and water stress conditions, a positive effect on plant survival is reported. Production of UV-B-induced flavonoids can be modulated by the application of UV-B prior or subsequent to water stress.

Adaptive responses to changing UV-B conditions play an important role in plant–herbivores interactions as well. UV-B-mediated changes in plant architecture, physiology, and/or chemistry can alter herbivorous arthropod’s performance and preference. In most cases, these UV-B-mediated induced physiological changes lead to the reinforcement of plant defenses. For example, increased production of UV-B-protective secondary metabolites and/or the reinforcement of plant cell walls induced by UV-B were proposed to affect plant colonization by herbivorous arthropods (Mazza et al., 1999, 2013; Rousseaux et al., 2004; Caputo et al., 2006; Foggo et al., 2007; Kuhlmann and Müller, 2010; Mewis et al., 2012; Zavala et al., 2015). However, in spite of the increasing literature of UV-B effects on plant–insect interactions, our current understanding is still hampered by the lack of an integrated approach that allows us to predict plant responses to diverse and changing environmental conditions. This aspect is of great importance when aiming for more environmental friendly agronomic practices and optimization of culture conditions. Modifications of plant chemistry and/or physiology by UV-B light can determine the responses of plants to other environmental variables, and vice versa (**Figure 1**). In natural conditions plants have to cope with constant variations in light intensity and quality, as well as with variations in abiotic factors such as increased temperature and reduced water availability. In some cases, responses to UV-B and to variations in these abiotic conditions converge to increase plant adaptation and, in addition, increase resistance to biotic stresses. However, antagonistic interactions between these responses may also occur and they may decrease plant defenses. Studies addressing these interactive effects on plant biochemistry and, eventually, the degree of resistance to arthropod herbivores are, however, lacking. In this review, we provide an overview on the existing knowledge on the single and interactive effects of UV-B and light quantity and quality, increased temperature and drought stress on plant growth and chemistry. We particularly focus on the possible implications for plant performance and protection against herbivorous arthropods.

## Effects of UV-B-Mediated Induced Secondary Metabolites on Plant Defenses Against Herbivores

Ultraviolet-mediated induction of phenolic compounds is one of the most common described plant responses that can directly alter the feeding of herbivorous insects. For instance, solar UV-B-mediated induction of the isoflavonoid glycosides daidzin and genistin in soybean (*Glycine max*) pods was reported to be negatively correlated with the percentage of damaged seeds by the stink bugs *Nezara viridula* and *Piezodorus guildinii* (Zavala et al., 2015). This was explained by the fact that isoflavonoids, a type of compounds restricted to plants of the Fabaceae family, are one of the main chemical defenses against herbivorous arthropods in soybeans. Also, chlorogenic acid, a phenolic acid induced by solar UV-B in *Nicotiana attenuata* (Ðinh et al., 2013) is reported to participate in plant defenses against insects. Oxidation of chlorogenic acid by plant polyphenol oxidases (PPOs) and peroxidases occurs after disruption of plant tissues caused by herbivory. This results in the production of highly reactive quinones that can covalently bind to leaf proteins and inhibit their digestion by the herbivore (War et al., 2012). Also, Ðinh et al. (2013) described that not only phenolic acids, but also the activity of defensive proteinase inhibitor proteins and levels of diterpene glucosides in *N. attenuata* plants were induced by solar UV-B. Interestingly, those authors demonstrated that the UV-B-mediated induction of a specific diterpene glycoside played a major role in *N. attenuata* defenses against the mirid *Tupiocoris notatus*. Hence, UV-B can modulate the production of different plant chemicals varying in their effects on plant resistance. Likewise, Mewis et al. (2012) described the UV-B-mediated induction of two different plant defense-related metabolites, flavonoids, and glucosinolates, in broccoli (*Brassica oleracea*) sprouts. This induction positively correlated with higher levels of resistance against the caterpillar *Pieris brassicae* and the aphid *Myzus persicae*. Glucosinolates produced by plants belonging to the order of Brassicales are nitrogen- and sulfur-containing glucosides that are hydrolyzed by myrosinases upon tissue disruption. The resulting hydrolyzed compounds, i.e., mainly isothiocyanates and nitriles, possess high toxicity against some herbivorous arthropods (Jeschke et al., 2015). However, whether UV-B-mediated induction of glucosinolates, alone or in combination with flavonoids, is responsible for the enhanced resistance against those herbivores has not been fully addressed. These examples highlight the complexity of the interactions between the UV-B-induced chemical defenses and herbivorous arthropods. Nevertheless, we can speculate that the overlapping plant responses to UV-B and herbivore’s attack might have a similar impact on plant defenses. For instance, this would be the case of common UV-B and herbivory-mediated induction of chlorogenic acid in *N. attenuata* plants (Izaguirre et al., 2007). In the same study, however, the flavonoid rutin was induced by UV-B, but not by herbivory. Increases in the levels of rutin, and also kaempferol derivatives, is a common response to UV-B in many plant species. Though these compounds have been reported to confer anti-herbivore properties, their role in plant defenses have been only addressed in a few studies, and these effects seem to depend on their concentration *in planta*. For instance, low rutin (quercetin-3-*O*-β-rutinoside) concentrations acted as phagostimulants to some polyphagous insects (e.g., *Schistocerca americana. Schistocerca albolineata*, and *Melanoplus differentialis*), but high concentrations deterred their feeding (reviewed by Simmonds, 2001). Yet, the degree of resistance given by an increase in these UV-B-induced compounds might also depend on the herbivore species. While increased susceptibility of *Arabidopsis* plants to the specialist caterpillar *P. brassicae* was associated with a significant reduction of kaempferol-3,7-dirhamnoside, no effect was observed for the specialist aphid *Brevicoryne brassicae* (Onkokesung et al., 2014).

In addition to the effect of UV-B on constitutive defenses (i.e., prior herbivore attack), UV-B has been demonstrated to alter the magnitude of the inducible plant defenses upon herbivory. When challenged by the feeding of arthropod herbivores, plants can perceive and display specific defense responses that are mainly regulated by the phytohormones jasmonic acid (JA), salicylic acid (SA), ethylene (ET), and abscisic acid (ABA) (Pieterse et al., 2012). Fine tuning plant defense responses is ultimately achieved by the cross-talk between JA, SA, ET, ABA, and other phytohormones. Activation of these signaling pathways is herbivore-species specific, and it leads to the production of defensive compounds such as secondary metabolites (e.g., alkaloids, glucosinolates, terpenes) and defensive proteins (e.g., proteinase inhibitors and PPOs) that deter herbivore’s feeding or alter its performance. In particular, activation of JA-associated defenses has been associated with increased resistance against leaf-chewing, piercing-sucking and some phloem feeding arthropods. In line with this, Ðinh et al. (2013) demonstrated that UV-B exposure of *N. attenuata* plants enhanced the JA burst and altered the accumulation of toxic 17-hydroxygeranyllinalool diterpene glycosides after infestation with the mirid *T. notatus*. Constitutive levels of JA, JA-isoleucine (JA-Ile) and ABA were not altered by the presence of solar UV-B, but herbivore-mediated induced JA defenses were augmented (i.e., primed) and, ultimately, plant resistance. Similarly, Demkura et al. (2010) demonstrated that UV-B-mediated induction of plant resistance to thrips (*Thrips tabaci* and *Frankliniella* spp.) in wild tobacco (*N. attenuata*) depended on the increased plant sensitivity to JA. Notably, though UV-B irradiated *N. attenuata* plants impaired in the JA pathway increased rutin and chlorogenic acid production, they did not display augmented resistance against thrips. This was explained by the necessary induction of the anti-herbivore PPOs, controlled by JA signaling, and whose preferred enzymatic substrate is chlorogenic acid. Therefore, the extent to which the plant’s chemical changes induced by UV-B confer antiherbivore properties can be highly related to the UV-B-mediated modulation of induced plant defenses.

## Interactive Effects of UV-B and Abiotic Factors on Plant Growth, Chemistry and Defenses Against Herbivores

### UV-B and Photosynthetically Active Radiation

Several studies have addressed the role of photosynthetically active radiation (PAR) (400–700 nm) in the modulation of plant sensitivity and photomorphogenic responses to UV-B radiation and vice versa. Direct, e.g., increased photorepair, photoreactivation and levels of photoprotective compounds, as well as indirect mechanisms, e.g., leaf anatomical changes, have been postulated to explain the UV protective effects of high PAR light conditions (Cen and Bornman, 1990; Deckmyn and Impens, 1997; Bolink et al., 2001; Krizek, 2004; Hoffmann et al., 2015). However, recent experimental evidence suggests that high PAR and UV-B might have a synergistic and positive effect on plant photoprotection. Prior exposure to UV-B has been shown to increase the net photosynthesis after subsequent exposition to high-light intensity conditions in lettuce (*Lactuca sativa*) (Wargent et al., 2011, 2015). Likewise, UV-B stimulated photosynthesis rates in Swedish ivy (*Plectranthus coleoides*) by increasing CO_2_ assimilation rate, stomatal conductance and internal CO_2_ concentration under high, but also low, PAR conditions (Vidović et al., 2015). Notably, under natural sunlight conditions, photo-inhibition (i.e., light-induced inactivation of photosystem II) in pumpkin (*Cucurbita pepo*) has been suggested to be caused by the UV-A, but not the UV-B component of solar radiation (Hakala-Yatkin et al., 2010). Moreover, this photoinhibitory effect is suggested to be attenuated by UV-B-inducible screens, i.e., accumulation of phenolic compounds in the plant epidermis.

Light intensity or distinct PAR levels have been demonstrated to influence the inducibility of plant responses triggered by contact with herbivore’s cues, but also to affect constitutive plant defenses. Gouinguené and Turlings (2002) showed that increasing light intensities positively correlated with increased volatile production in herbivore- induced corn (*Zea mays*) plants. This might be correlated with an increase in the hormone-signaling involved in these defense responses. For instance, an enhanced generation of JA precursors has been described under high light conditions (Frenkel et al., 2009). In tomato, constitutive levels of defensive leaf trichome densities and their associated allelochemicals were induced by increased light intensity, which correlated with augmented resistance against the caterpillar *Manduca sexta* (Kennedy et al., 1981). Furthermore, high PAR has been reported to induce other leaf secondary metabolites, such as flavonoids and phenolic acids, which might affect plant–insect interactions. A synergistic effect in the production of these compounds is often reported when both high PAR and UV-B irradiance is applied to plants (Götz et al., 2010; Guidi et al., 2011; Barnes et al., 2013; Müller et al., 2013; Vidović et al., 2015). This suggests a common acclimation response of plants to both light signals (Wargent et al., 2015) and, therefore, also their possibly positive effect on plant defenses against biotic stresses. Interestingly, when both high PAR and UV-B irradiances are applied, a greater increase in the concentration of flavonoids is detected in old plant leaves. For instance, while high PAR induced the accumulation of flavonoids in young leaves of barley (*Hordeum vulgare*) plants, a combined treatment with high UV-B increased the production of these compounds in older leaves as well (Klem et al., 2012). Similarly, higher flavonoid production in young, but also in older leaves of silver birch (*Betula pendula*) plants grown under ambient PAR and UV-B have been reported (Morales et al., 2013). The fact that older leaves experience an increase in the content of phenolic compounds might have repercussions for plant protection against herbivores. Some herbivore arthropods show a high feeding and oviposition preference for older parts of the plants over the young ones. Some examples are the whitefly *Bemisia tabaci* (Zhang and Wan, 2012) and the thrips *Frankliniella fusca* on tomato (*Solanum lycopersicum*) (Joost and Riley, 2008), *F. occidentalis* on *Senecio* hybrids (*Senecio jacobaea* × *Senecio aquaticus*) (Leiss et al., 2009) and tomato (Mirnezhad et al., 2010), and the larvae of *Spodoptera litura* on radish (*Raphanus sativus*) (Yadav et al., 2010). We can, therefore, speculate that an encounter of the herbivore with better protected old leaves might negatively impact their performance and/or survival.

Besides the enhancement of constitutive defenses in older parts of the plant by combined high PAR and UV-B conditions, it remains unknown whether this positive effect also extends to an increase in the capacity of older plant parts to respond to herbivore’s attack. Old plant leaves are reported to be less responsive to herbivore-mediated induced defenses, which might affect direct and indirect (i.e., attraction of natural enemies of the herbivore) defense responses. For example, the predator *Phytoseiulus persimilis* was reported to be attracted to the emitted-volatiles of spider mites-infested young leaves of cucumber plants, but less to infested old leaves (Takabayashi et al., 1994). These indirect induced defenses are controlled by JA and SA signaling pathways (Ament et al., 2004). A higher induction of these defenses in young leaves with respect to older ones might explain these differences. As UV-B can prime JA-mediated induced defenses against insects, we might hypothesize that combined high PAR and UV-B conditions do not antagonize each other, but they rather might have a positive and/or synergistic effect on these inducible plant defenses. This is an aspect that needs further research.

### UV-B and UV-A

Ultraviolet-A (315–400 nm) constitutes the major component of the solar UV spectrum. Plants perceive and respond to UV-A by inducing photomorphogenic responses that, in some cases, resemble those triggered by UV-B. For example, stem elongation and leaf enlargement were decreased under ambient UV-A in cucumber (*Cucumis sativus*) (Krizek et al., 1997) and lettuce (Krizek et al., 1998). Interestingly, UV-A can interact with UV-B to modulate plant responses. For instance, UV-A can mitigate the deleterious effects of UV-B on the photosynthetic apparatus under low PAR conditions (Adamse et al., 1994), as demonstrated in barley (Štroch et al., 2015, cluster bean (*Cyamopsis tetragonoloba*) (Joshi et al., 2007, 2013) and the woody shrub *Pimelea ligustrina* (Turnbull et al., 2013).

In contrast to the well-known effects of UV-B on plant–insect interactions mediated by changes in plant quality, the role of UV-A has not been well explored so far. The effects of UV-A on constitutive chemical defenses, however, can differ from those induced only by UV-B. For instance, higher accumulation of epidermal flavonoids was not stimulated by UV-A, but by a combined UV-A and UV-B treatment in silver birch (Morales et al., 2010, 2011) and *Arabidopsis* (Morales et al., 2013). These results suggested a major role of UV-B on the induction of flavonoids. However, UV-A has been described to modulate the UV-B associated responses in plants. For instance, in turnip hypocotyls, while both UV-A and B induced anthocyanin biosynthesis, the pattern of anthocyanin accumulation along the hypocotyl greatly differed depending on the wavelengths of UV applied (Zhou et al., 2007; Wang et al., 2012). Also, Morales et al. (2010) described different changes in the abundance of specific flavonoids when UV-A or UV-B were depleted. Under exclusion of UV-B, young silver birch leaves accumulated less of six epidermal flavonoids (i.e., myricetin-3-galactoside, quercetin-3-galactoside, quercetin-3-rhamnoside, and kaempferol-3-rhamnoside), while UV-A exclusion decreased the accumulation of only quercetin-3-galactoside and quercetin-3-arabinopyranoside. Likewise, Wilson et al. (2001) reported that UV-A reduced the production of UV-B-inducible flavonoids in rape by shifting the abundance of particular quercetin compounds. A common regulatory component of plant responses to both types of UV was therefore proposed. In particular, Morales et al. (2013) suggested that the UV-photoreceptor UVR8 may be involved in the UV-A regulation of individual metabolites in *Arabidopsis*. This was supported by the necessary activation of UVR8 for UV-A induction of kynurenic and chlorogenic acids, tryptophan, phenylalanine, kaempferol and kaempferol-3-rhamnoside in *Arabidopsis* (Morales et al., 2013). How these UV-A and UV-B interactions might affect plant responses to herbivory is still unknown. However, we might hypothesize that changes in the abundance of plant specific phenolics may alter the feeding behavior of herbivorous arthropods. This might be illustrated by the experiments performed by Hamamura et al. (1962). These authors demonstrated that while the quercetin-3-*O*-glucoside acted as a feeding stimulant for the silkworm (*Bombyx mori*) in white mulberry (*Morus alba*) leaves, another quercetin glucoside, quercetin-3-*O*-rhamnoside, deterred larval feeding, and 3-*O*-rutinoside did not have any effect at all.

### UV-B and Blue Light

Blue light (400–500 nm) regulates diverse plant processes such as phototropism, photomorphogenesis, stomatal opening, and leaf photosynthetic functioning (Whitelam and Halliday, 2008). During plant growth it constitutes an essential part of the development of higher plants. For instance, increasing levels of supplemental blue light are positively correlated with leaf photosynthesis, even under low light irradiances in cucumber (Hogewoning et al., 2010). Under red light conditions, it has been reported that supplemental blue light can enhance dry matter production in radish, lettuce, and spinach (*Spinacia oleracea*) (Yorio et al., 2001; Johkan et al., 2010), as well as leaf photosynthesis in pepper (Brown et al., 1995) and rice (*Oryza sativa*) (Matsuda et al., 2004). Yet, the intensity of combined red and blue light conditions was suggested to determine the energy efficiency and the net photosynthesis rate in tomato (Fan et al., 2013).

Supplemental blue light in plants prior to, simultaneously with, or subsequent to UV-B exposure, can prevent the damaging effects of high UV-B radiation, therefore showing certain similarities with the effects described for high PAR. For instance, blue light (i.e., 62% of PAR) increased the acclimation of pepper and cucumber plants to UV radiation under high light intensity conditions (Adamse et al., 1994; Hoffmann et al., 2015). This has been explained by a lower degradation of photosynthetic related pigments (chlorophyll a and b, and carotenoids) by UV (Hoffmann et al., 2015), as well as the increase in epidermal flavonols when plants were grown under enriched blue light radiation (Adamse et al., 1994; Ebisawa et al., 2008; Son and Oh, 2013; Hoffmann et al., 2015; Ouzounis et al., 2015; Siipola et al., 2015). These observations have led to some authors to propose blue light as the major constituent of the sunlight responsible for the upregulation of the epidermal content of flavonoids (Ouzounis et al., 2014; Siipola et al., 2015). If so, its importance in the reinforcement of plant defenses against herbivorous arthropods might be highly overlooked. In line with this, diminished blue light was reported to reduce the accumulation of quercetin derivatives in apical, cauline and basal leaves of pea (*Pisum sativum*) (Siipola et al., 2015) which, as discussed previously, might influence plant protection against herbivores in older and, therefore, more susceptible plant leaves. Also, the distribution of flavonoid compounds in the plant under different solar/blue radiation might not only differ in young and old leaves, but also within the leaf cell layers. For example, flavonoid accumulation in shade leaves of the green olive tree (*Phillyrea latifolia*) has been reported to occur mainly in the adaxial epidermal layer. However, in sun leaves of this tree flavonoids also accumulated in sub-epidermal cells leading to a steeper gradient in flavonoid concentration from the adaxial epidermis to the inner spongy layers (Tattini et al., 2000; Agati et al., 2002). A deeper distribution of these compounds within the plant leaf might alter the performance of herbivores that feed preferentially on the mesophyll cell layers while avoiding the epidermis, such as the larvae of leaf miners (Sinclair and Hughes, 2010) or cell-content feeders as thrips (Chisholm and Lewis, 1984) and spider mites (Helle and Sabelis, 1985).

Though plant perception and responses to blue and UV-B light have been addressed in several studies (see review by Huché-Thélier et al., 2016), their interactive effects on feeding and/or survival of herbivorous arthropods have not been investigated so far. Yet, the similarities in the plant responses triggered by both light signals suggests that the positive UV-B effect on plant defenses against herbivores might not be counteracted by blue light, but the contrary. Supporting this hypothesis, a synergistic effect between blue and UV-B on the production of UV-B-induced flavonoids has been reported to occur under both light irradiances. This is the case of the production of anthocyanins, which was significantly enhanced under combined blue light and UV-B radiation conditions in the hypocotyls of turnip seedlings (Wang et al., 2012). This might be explained by the reported synergy in the induction of key genes involved in flavonoid biosynthesis such as *chalcone synthase* (*CHS*) in *Arabidopsis* (Fuglevand et al., 1996*;* Wade et al., 2001) and turnip (*Brassica rapa*) (Wang et al., 2012), as well as *flavonol synthase* in lettuce (Ebisawa et al., 2008), under combined blue and UV-B light conditions. However, whether these transcriptomic responses also extend to augmented plant responses against herbivorous arthropod is still unknown.

### UV-B and Far-Red Light

Far-red (FR) light (700–780 nm) modulates a wide range of physiological responses in plants. Higher FR radiation or low-Red (R):FR ratios, resulting from shade conditions, constitute a signal of competition for light in dense plant canopies (Ballaré, 1999). In shade-avoidance species, such as *Arabidopsis*, typical plant responses to low R:FR ratios are principally regulated by the phytochrome B and include hyponasty (i.e., more vertical orientation of the leaves) and enhanced stem and petiole elongation (reviewed by Pierik and de Wit, 2013).

In general, plant morphological and biochemical features under low R:FR conditions have been associated with weaker defense responses against herbivores (Izaguirre et al., 2006; Kegge et al., 2013). For instance, in FR-supplemented tobacco (*N. longiflora*) plants, the caterpillar *M. sexta* grew faster than on ambient light-treated plants (Izaguirre et al., 2006). Likewise, growth of *S. frugiperda* caterpillar in *Arabidopsis* was increased when plants were grown under enriched FR conditions (Moreno et al., 2009). This was explained by the R:FR- mediated suppression of inducible plant defenses controlled by the JA and SA defense-related hormone signaling pathways (Wit et al., 2013; Radhika et al., 2010). However, constitutive defenses can be also affected. For instance, Cortés et al. (2016) recently demonstrated that density of defensive trichomes was reduced in the stems of tomato mutants defective in the perception of red light by the phytochrome B. Moreover, these authors also reported lower concentration of leaf flavonoids in the tomato mutants. In line with this, inactivation of phytochrome B by low R:FR ratios has been shown to negatively regulate induction of *CHS* in *Arabidopsis* (Wade et al., 2001).

Recently, it has been demonstrated that UV-B perception by plants blocks the signal transduction triggered by low R:FR conditions (Hayes et al., 2014; Mazza and Ballaré, 2015). These results agree with a previous study by Tegelberg et al. (2004) showing that under combined supplemental UV-B and FR light conditions, concentrations of quercetins, kaempferols, and chlorogenic acid were increased by UV-B irrespective of the FR treatment in silver birch seedlings. However, UV-B-mediated induction of plant flavonoids can depend on the FR doses plants are exposed. Gerhardt et al. (2008) observed a suppression of UV-B-mediated induction of flavonoids in FR light pre-irradiated *B. napus* plants when increasing the amount of FR in the spectrum. These authors also described that under supplemental UV-B and moderate levels of FR, higher contents of kaempferol glycosides were detected, while levels of quercetin glucosides were reduced. Overall, these results suggest that the magnitude of the UV-B or FR light signal can determine the outcome of plant chemical responses and, therefore, constitutive plant defenses against herbivores. In addition, as UV-B has been shown to increase plant responsiveness to JA-defenses, a negative trade-off between plant responses to low R:FR ratios and UV-B light might be expected. However, whether the negative effects of low R:FR ratio on plant defenses can be neutralized by the positive impact on constitutive and/or inducible plant defenses of UV-B is an aspect that needs further research.

### UV-B and Increased Temperature

Temperature is a key parameter regulating many processes of plant physiology. Increases in temperature are reported to lead to increased plant growth, as an effect of enhanced photosynthetic rates (Tollenaar, 1989; Saxe et al., 2001; Nybakken et al., 2012). Though there are many studies describing the effect of temperature and UV-B on plants separately, there is limited research on their combined effects on plant physiology and/or chemistry. Most of the these studies, however, report a compensatory effect of enhanced temperature on UV-B-mediated inhibition on plant growth. In studies with sunflower (*Helianthus annuus*) and maize (*Zea mays*), for instance, Mark and Tevini (1996) showed that an increase in temperature (28–32°C) resulted in higher values of absolute growth parameters regardless of UV-B treatment. Moreover, these authors described that higher temperature compensated the negative effect of UV-B on plant growth. Similar findings were reported by Han et al. (2009) for dragon spruce (*Picea asperata*) seedlings. Enhanced UV-B reduced growth, chlorophyll content and net photosynthesis rate, but these effects were alleviated by higher temperature. On the other hand, pre-exposition to low and ambient doses of UV-B promoted heat tolerance in cucumber (Teklemariam and Blake, 2003) and conifer seedlings (L’Hirondelle and Binder, 2005).

Temperature is a very important factor affecting herbivore performance directly (Bale et al., 2002), or indirectly by altering the host plant quality (Grinnan et al., 2013). For instance, temperature-dependent changes in primary and secondary plant metabolites were suggested to explain the negative effects of increased temperature (17–25°C) on *Pieris napi* larvae development on *Sinapis alba* (Bauerfeind and Fischer, 2013). Enhanced temperature conditions, however, have shown to decrease the content of phenolic compounds in dark-leaved willow (*Salix myrsinifolia*) (Veteli et al., 2002; Paajanen et al., 2011; Nybakken et al., 2012) and Norway spruce (*Picea abies*) (Virjamo et al., 2014). This negative regulation of plant phenolics is most probably caused by the temperature-dependent regulation of genes involved in their biosynthesis. Transcript levels of essential regulators of flavonoids in *Arabidopsis* are reported to strongly increase under decreasing temperatures (Olsen et al., 2009; Dao et al., 2011). Furthermore, a slower degradation of quercetin and kaempferol glycosides has been described under lower temperatures (Olsen et al., 2009). Under both UV-B and enhanced temperature the negative effect on plant phenolics persists in dark-leaved willow plants, where combined increased temperature (2°C above ambient) and UV-B decreased the content of phenolic compounds (i.e., chlorogenic and cinnamic acids) (Nybakken et al., 2012). In addition to phenolic acids, tannins have been reported to be negatively affected by the interaction between temperature and UV-B. For instance, in European aspen (*Populus tremula*) seedlings, Randriamanana et al. (2015) observed a genotype-specific reduction of soluble condensed tannins when both UV-B and temperature (13.7–24°C) were augmented. In the same study, flavonoid production was induced by enhanced UV-B, but diminished by increased temperature. Nevertheless, there are some studies in the literature that did not describe negative interactions between enhanced temperature and UV-B on plant phenolics (Lavola et al., 2013; Neugart et al., 2014). Lavola et al. (2013) described that UV-B increased the accumulation of condensed tannins, quercetin derivatives, rhamnosylated kaempferol and phenolic acids in silver birch seedlings, but these compounds were not affected by elevated temperature (21.9–24.4°C) (Lavola et al., 2013). This might be explained by a lower responsiveness of flavonoid biosynthesis-related genes when variations in temperature occur in that specific range (Olsen et al., 2009). Still, how UV-B and temperature interact at the transcriptional level regulating the production of phenolic compounds is an aspect that has not been investigated yet.

As previously described, flavonoids might determine plant resistance against herbivorous arthropods, and a reduced UV-B-mediated induction of these compounds might influence these interactions. However, condensed tannins, in turn, are not clearly associated with negative effects on insect herbivore performance (reviewed by Barbehenn and Constabel, 2011). Only a few studies have reported negative correlations between the presence of condensed tannins and insect feeding and/or performance. Moreover, higher production of condensed tannins was reported to increase caterpillar feeding in transgenic hybrid aspen (*Populus tremula* × *tremuloides*) (Boeckler et al., 2014) and thrips damage in poplar (Mellway and Constabel, 2009). Tannins can precipitate proteins only at low pH. While the gut of vertebrate animals has a low pH, the gut of many arthropods is highly alkaline. Tannins might also be oxidized in the guts of insects, generating quinones that might link to proteins and make them non-digestible for the insect. However, recent reports showed that condensed tannins are the least oxidatively active, and that some condensed tannins even inhibit the pro-oxidant activity of ellagitannins (hydrolysable tannins) (reviewed by Salminen and Karonen, 2011). We can, therefore, consider that a reduction in condensed tannins might not have direct effects on plant–insect interactions. However, Madritch and Lindroth (2015) recently showed that variation in condensed tannin concentration is correlated with plant nitrogen recovery following a severe defoliation event, such as that caused by herbivory. From this point of view, reduced condensed tannins might influence plant tolerance to insect herbivores, i.e., by reducing re-growth capacity, rather than defense.

Though it seems that increased warming might cause a reduction in plant protection against herbivorous arthropods by diminishing the UV-B-mediated accumulation of phenolics, other defense-related compounds are reported to increase under incremental temperatures. Emission of volatile organic compounds (VOCs) can be positively influenced by temperature, as reported for monoterpenes in sunflower and beech trees (*Fagus sylvatica*) (Schuh et al., 1997), and for the sesquiterpene β-caryophyllene in orange trees (*Citrus sinensis*) (Hansen and Seufert, 2003). Besides protecting plants from abiotic stresses, plant volatiles are crucial in plant–arthropod interactions. For instance, the sesquiterpene β-caryophyllene has been described as a key component in the attraction of plant enemies to insect infested-maize plants (Köllner et al., 2008). Interestingly, a positive interaction between UV-B and enhanced temperature on VOCs production has been recently described by Maja et al. (2016). They reported higher isoprene emissions in European aspen under enhanced UV-B radiation (31% above ambient) but only in combination with increased temperature (ambient +2°C) conditions. Isoprene is the dominant VOC released to the atmosphere by vegetation, though not emitted by all plant species (Kesselmeier and Staudt, 1999). Apart from its antioxidant capacity, isoprene can mediate plant–herbivore interactions. For instance, Laothawornkitkul et al. (2008) demonstrated that the emission of isoprene in tobacco (*N. tabacum*) deterred feeding of *M. sexta* caterpillars. The ecological importance of these interactions for plant–insect interactions, however, remains to be determined.

### UV-B and Drought Stress

The interrelation between drought stress and UV-B, and their combined action on plant physiology has been amply studied (see Bandurska et al., 2013, for review). When moderate UV-B and drought conditions occur simultaneously or sequentially, both can interact synergistically to increase plant tolerance and, consequently, plant survival. These responses were associated to enhanced production of antioxidant proteins, UV-B absorbing compounds and higher leaf cuticle thickness, among others. However, less is known about the outcome of drought stress and UV-B interactions on the production of plant chemical defenses and their effects on plant–herbivore interactions.

Drought stress has been shown to greatly influence plant resistance and defense responses against herbivores (see review by Foyer et al., 2016). However, these drought-induced plant responses have shown no clear pattern in their effects on insects. Though some phytophagous insects benefit from water-stressed hosts (Mewis et al., 2012; Tariq et al., 2013), this abiotic stress can also interact negatively with the performance of herbivorous arthropods (Nguyen et al., 2016; Pineda et al., 2016). Positive effects might be explained by the increased content of amino acids and soluble sugars (Mewis et al., 2012). In turn, negative effects have been proposed to be explained by the reduction in turgor pressure, water content, plant growth, and higher concentration of allelochemicals. For instance, flavonoids and anthocyanins have been reported to accumulate in wheat (*Triticum aestivum*) (Ma et al., 2014), and soluble phenols in pea (Alexieva et al., 2001), under drought stress conditions. Interestingly, constitutive levels of glucosinolates have been reported to be affected by drought stress as well. For instance, Mewis et al. (2012) described increased levels of flavonoids and glucosinolates in drought-stressed *Arabidopsis* plants. In the same study, however, the generalist aphid *M. persicae* performed better in plants subjected to drought, which suggested that induction of glucosinolates did not have a great impact on these interactions. Conversely, Pineda et al. (2016) described that drought conditions decreased the population growth of *M. persicae* in *Arabidopsis*. These authors also showed that this negative effect was maintained in *Arabidopsis* mutants defective in the production of glucosinolates, suggesting the existence of other mechanisms involved in these interactions. However, whether other drought-induced secondary metabolites had a role in *Arabidopsis* resistance to aphids was not further investigated. In this regard, not only phenolic compounds, but higher terpene and benzenoid emissions have been described for some plant species subjected to drought stress or combined drought and herbivory (Copolovici et al., 2014; Weldegergis et al., 2015). Mono- and sesquiterpenes can protect plant membranes against peroxidation and water stress-induced reactive oxygen species by acting as potent antioxidants (Tattini et al., 2015), but they also are important mediators in the interactions of plants with herbivores and the herbivore’s natural enemies. Hence, the blends of volatiles released by herbivore-infested plants provide natural enemies cues to locate their prey. In line with this, though the effect of drought-mediated induction of plant volatiles on direct plant responses against herbivores has not been clarified yet, their possible impact on indirect plant defenses was recently explored. Weldegergis et al. (2015) showed that drought stress augmented the emission of volatile compounds in *Mamestra brassicae*-infested *B. oleracea* plants. They described that the parasitic wasp *Microplitis mediator* showed equal preference for plant volatiles emitted from *M. brassicae*-damaged plants and plants exposed to combined drought and herbivory. In another study, however, Tariq et al. (2013) reported that plants exposed to both root herbivory and drought negatively affected the preference of a aphid parasitoid for aphid-infested plants. This was explained by the modification of the aphid-mediated induced volatile blend in simultaneously drought stressed- and root feeder-infested plants.

The interactive effects of drought and UV-B on the production of secondary metabolites do not show a clear pattern. Prior treatment with high ratio of UV-B to PAR treatment enhanced the production of flavonoids in pea plants that were subsequently subjected to drought conditions (Nogués et al., 1998). Conversely, simultaneous enhanced UV-B treatment and drought stress dramatically reduced the UV-B-mediated induction of anthocyanins and flavonols in barley (Bandurska et al., 2012) and pea (Alexieva et al., 2001). These contrasting effects might be explained by different experimental conditions, i.e., different levels of drought stress, but also by plant-species specific responses. Regarding VOCs, only a few studies have addressed the effect of combined UV-B and water stress on VOCs emission. Some authors have described that UV-B can alter emissions or increase endogenous leaf accumulation of VOCs (Tiiva et al., 2007; Llusia et al., 2012; Alonso et al., 2015). When combined with drought, however, Alonso et al. (2015) reported lower UV-B-mediated induction of terpene compounds in grapevine (*Vitis vinifera*). Conversely, Llusia et al. (2012) showed that terpene emissions were altered by increased UV-B and water stress in a species-specific manner in Mediterranean species of xerophytes (*Daphne gnidium*, and *Pistacia lentiscus*) and mesophytes (*Ilex aquifolium* and *Laurus nobilis*). While in one of these species UV-A+B increased terpene emissions, water stress only had a positive effect in another species, and combined water stress and UV-A+B conditions elicited a stronger response. In summary, though drought and UV-B can strongly modulate plant constitutive defenses through changes in secondary metabolites, more effort is needed to elucidate the impact of these chemical changes in plant resistance against herbivores.

In addition, whether the single effects of drought or UV-B on plant induced defenses against herbivores can differ from the plant responses triggered by simultaneous drought and UV-B is an aspect that needs to be elucidated. Remarkably, drought can increase JA accumulation and JA-induced defenses in plants (Nguyen et al., 2016), suggesting that combined drought and UV-B effects on plant responses to insect herbivores might not neutralize each other.

### Future Perspectives

Implementation of systems that can modulate UV-B irradiances in greenhouses to increase crop protection against pests is promising. However, modern agriculture is highly dependent on other environmental variations (Mittler and Blumwald, 2010). First, environment conditions have a high influence on plant growth and yield and, second, they influence the outbreaks of crop pests and how plants respond to these attackers. Thus, agricultural systems located in different areas of our planet have to face different climate challenges. Adaptation to these diverse environments requires knowledge to predict the outcome of crop production when UV-B is applied and, accordingly, to implement measures that can benefit plant’s performance. For instance, the agricultural challenges in higher altitude and temperate regions differ greatly from those in tropical and subtropical zones. While in the first case limited solar radiation and low temperatures determine agronomy’s practices, in the second high PAR, high temperature, high UV-B radiation, reduced water availability and increasing pest’s outbreaks are the principal challenges for the sustainability of crops. In middle and higher latitude regions, the cold conditions reduce the survival of arthropod pests, but in order to increase the productivity under limited light and low temperatures the maintenance costs of the greenhouse are higher. Importantly, knowledge on light perception and responses by plants to the interactive effects of UV-B light with other abiotic conditions can help to optimize culture conditions. For instance, understanding the effects of UV-B:PAR ratio on plant chemistry has been shown to be fundamental to avoid plant stress, and to promote desirable photomorphogenic responses (reviewed by Wargent and Jordan, 2013). This is of special importance in greenhouses installed in higher latitudes of temperate areas, where low PAR levels during winter require the use of supplemental light systems (see review by Vänninen et al., 2010).

In warmer areas, pest’s outbreaks have a predominant influence on crops productivity. In order to increase protection against pests, traditional greenhouses are generally built with plastic materials that also block the transmission of UV-A and -B light. Lack of UV-B, but specially UV-A, blocks the orientation of some insects inside the greenhouse, such as thrips and whiteflies, which can result in reduced plant damage and transmission of virus diseases (Mazza et al., 1999; see also review by Johansen et al., 2011). These responses, however, are species-specific, and UV-B has been demonstrated to induce avoidance responses in other arthropod pests. Such is the case of the spider mite *Tetranychus urticae* (Barcelo, 1981). Hence, we can speculate that the use of UV-B-transmitting films might be beneficial in areas where this pest is predicted to experience strong upsurges. Though discussion of direct effects of UV-B and other biotic factors on behavior of herbivorous arthropods is not the main goal of this review, their interactions cannot be overlooked when aiming for integrated pest management practices. Increasing evidence presented here, however, suggests that the beneficial effects of UV-B on plant physiology and resistance to pests might also confer as much benefits as the exclusion of this UV light signal from the greenhouse environment.

Ultraviolet-B can interact positively with high PAR, blue light, temperature and water stress to increase plant performance and constitutive chemical defenses. Under mild water stress, for instance, the use of UV-B-transmitting films might alleviate the stress responses of crop plants (Bandurska et al., 2013). Water stress responses are, in some cases, associated with plant susceptibility to herbivorous arthropods. Whether UV-B can ameliorate the negative effects of water restrictions on plant resistance would require investigating different aspects of plant–insects interactions. First, how changes in constitutive defenses alter the insect’s preference and performance in the host plant. Second, how inducible plant defenses controlled by plant’s hormone signaling are modulated by drought and UV-B. The use of mutant plants deficient in constitutive production of secondary metabolites and/or in herbivore-mediated induced defenses can shed light on the mechanisms operating in UV-B and abiotic factors interactions. Also, to unravel these complex interactions the use of different experimental settings is required. The use of growth cabinets for UV-B supplemental or exclusion experiments exposes plants to unrealistic conditions when compared to greenhouse environments. However, their controlled conditions provide some advantages, as they facilitate the specific assessment of interactions between abiotic factors, and to determine the mechanisms behind. Then, assessment of these effects under greenhouse conditions would be a further step to verify the implementation of new agronomic practices. Remarkably, current literature on the effect of UV-B on plant–insect interactions has mainly focused on a few model plants and crop species, such as *A. thaliana, N. attenuata*, broccoli (*B. oleracea*) and soybean (*G. max*). No studies on the effect of UV-B on plant resistance against herbivores have been described for economically important crop species such as tomato, and less is known about the interactive effects of UV-B and abiotic factors on these interactions. Additionally, how different varieties of the same crop differ in their responses to changing UV-B and abiotic conditions such as drought, heat and light intensity/quality, and how these affect their capacity to protect against major pests is a another question that needs to be investigated.

One of the major limitations in agricultural systems is the area crops plants have available to grow. This leads to reduction in the inter-plant distances, and results into crowded plant canopies. As a consequence, this environment is enriched in far-red and deficient in red light, which promotes shade-avoidance responses in plants (see review by Ballaré, 2009). Herbivorous arthropods take advantage of this situation, as they find better shelter to escape from natural enemies, protection from direct UV-B damaging effects, and weaker plant defenses. As described here plant responses to UV-B and low-Red:far-red ratios are reported to counteract each other. We hypothesize that implementation of supplemental UV-B within plant canopies might constitute a promising alternative to increase crop protection against pests. Moreover, UV-B-mediated reinforcement of plant defenses against herbivores can increase plant yield in the absence of pesticides, as demonstrated by Mazza et al. (2013) in soybean (*G. max*). Though negative trade-offs between plant growth and defenses are generally the rule, UV-B-mediated reinforcement of constitutive and inducible plant defenses against herbivores might optimize the usage of plant resources. In other words, UV-B-induced production of secondary metabolites can inhibit colonization of insects, thus reducing the energy investment to replace the plant tissues consumed by herbivores (Karban, 2011). Similarly, UV-B-mediated priming of inducible plant defenses, which results in stronger and faster defense responses could stop herbivore infestations at earlier stages, reducing the negative effects on plant growth and yield (Frost et al., 2008). In this regard, it would be interesting to investigate whether a blue light-enriched environment might optimize these UV-B-mediated plant responses. As blue light can increase the photosynthetic capacity of plants, the availability of substrates for production of secondary metabolites might be augmented. Nevertheless, this is an aspect that needs further research.

In summary, modulation of UV-B light in agriculture systems constitutes a promising tool to increase crop production and protection against pests. However, the inherent complexity of the UV-B-mediated effects on plant–herbivore interactions when the crosslink effects of different abiotic conditions are considered demands for a better understanding of plant responses to these changing environment conditions.

## Author Contributions

RE-B wrote the first concept of the manuscript and made **Figure 1**. PK and KL provided ideas and discussions points and contributed to the final manuscript.

## Conflict of Interest Statement

The authors declare that the research was conducted in the absence of any commercial or financial relationships that could be construed as a potential conflict of interest.
